# Simulating Cross-Contamination of Cooked Pork with *Salmonella enterica* from Raw Pork through Home Kitchen Preparation in Vietnam

**DOI:** 10.3390/ijerph15102324

**Published:** 2018-10-22

**Authors:** Sinh Dang-Xuan, Hung Nguyen-Viet, Phuc Pham-Duc, Delia Grace, Fred Unger, Nam Nguyen-Hai, Thanh Nguyen-Tien, Kohei Makita

**Affiliations:** 1 Center for Public Health and Ecosystem Research, Hanoi University of Public Health, 1A Duc Thang Road, Duc Thang Ward, North Tu Liem District, Hanoi 100000, Vietnam; dxs@huph.edu.vn (S.D.-X.); H.Nguyen@cgiar.org (H.N.-V.); pdp@vohun.org (P.P.-D.); nam.global247@gmail.com (N.N.-H.); 2 Veterinary Epidemiology Unit, School of Veterinary Medicine, Rakuno Gakuen University, 582 Bunkyodai Midorimachi, Ebetsu, Hokkaido 069-8501, Japan; 3 International Livestock Research Institute, 298 Kim Ma Street, Hanoi 100000, Vietnam; F.Unger@cgiar.org; 4 Department of Epidemiology and Public Health, Swiss Tropical and Public Health Institute (Swiss TPH), 57 Socinstrasse, 4002 Basel, Switzerland; 5 University of Basel, Socinstrasse 57, 4002 Basel, Switzerland; 6 International Livestock Research Institute, 30709 Naivasha Street, Nairobi 00100, Kenya; D.GRACE@cgiar.org; 7 National Institute of Veterinary Research, 86 Truong Chinh, Hanoi 100000, Vietnam; ntt3180@gmail.com

**Keywords:** cross-contamination, pork, *Salmonella enterica*, simulation, Vietnam

## Abstract

Pork is the most commonly consumed meat in Vietnam, and *Salmonella enterica* is a common contaminant. This study aimed to assess potential *S. enterica* cross-contamination between raw and cooked pork in Vietnamese households. Different scenarios for cross-contamination were constructed based on a household survey of pork handling practices (416 households). Overall, 71% of people used the same knife and cutting board for both raw and cooked pork; however, all washed their hands and utensils between handling raw and cooked pork. The different scenarios were experimentally tested. First, *S. enterica* was inoculated on raw pork and surfaces (hands, knives and cutting boards); next, water used for washing and pork were sampled to identify the presence and concentration of *S. enterica* during different scenarios of food preparation. Bootstrapping techniques were applied to simulate transfer rates of *S. enterica* cross-contamination. No cross-contamination to cooked pork was observed in the scenario of using the same hands with new cutting boards and knives. The probability of re-contamination in the scenarios involving re-using the cutting board after washing was significantly higher compared to the scenarios which used a new cutting board. Stochastic simulation found a high risk of cross-contamination from raw to cooked pork when the same hands, knives and cutting boards were used for handling raw and cooked pork (78%); when the same cutting board but a different knife was used, cross-contamination was still high (67%). Cross-contamination between was not seen when different cutting boards and knives were used for cutting raw and cooked pork. This study provided an insight into cross-contamination of *S. enterica*, given common food handling practices in Vietnamese households and can be used for risk assessment of pork consumption.

## 1. Introduction

Foodborne diseases (FBD) are a major health problem and contribute to reduced economic productivity [1,2]. The first global assessment found the health burden of FBD was comparable to that of malaria, tuberculosis or HIV/AIDS [2]. In Vietnam, 1781 food poisoning outbreaks were reported between 2006 and 2015 affecting 58,622 people and causing 412 deaths [3]. The actual number of cases is likely to be far higher as under-reporting of FBD is common [4].

Pork and pork products are the most commonly consumed meat in Vietnam [5]. They are also an important source of *Salmonella*, second to eggs and poultry meat [4,6,7]. In Vietnam, most pork (80%) is produced by smallholders and sold in informal markets (open air with limited infrastructure and no cold chain) [8]. Swine commonly harbor *Salmonella* without showing clinical signs [9,10,11] and are one of the main reservoirs of human salmonellosis. Studies in Vietnam have found *Salmonella* in the feces of apparently healthy pigs, with one study reporting a prevalence of 5.2% [12]. Other studies have reported prevalence ranging from 38.9% to 49.4% in feces collected at slaughterhouses [13,14]. Reported prevalence on carcasses in slaughterhouses in Vietnam varies from 15.5% to 95.7% [14,15,16,17]. The prevalence of *Salmonella* on pork products sold in wet markets in Vietnam ranges from 32.8% to 69.9% [14,16,18,19]. The general increase in prevalence along the chain suggests that cross-contamination occurs, and a report suggests that 46.7% of cross-contamination occurs during transportation to the market, which is often un-hygienic (e.g., closed containers are not used, and transport is not clean) and at the market itself [17].

Information on the prevalence of *S. enterica* in household pork in Vietnam is not available, partly due to ethical and practical challenges with collecting data. During food handling and preparation at home, microorganisms in raw foods can be transferred directly from hands, other surfaces, equipment or utensils to cooked food [20]. Household food preparation may be riskier than commercial food establishment preparation, due to less hygienic handling [21].

Microbiological food safety risk assessment is a powerful tool to understanding the magnitude of health risks from pathogenic bacteria present in foods [22,23]. As part of this, transfer experiments can provide data allowing risk assessment steps to be better described and modeled [24]. This study was designed to gain knowledge about levels of *S. enterica* transferred during pork preparation in home kitchens and support a risk assessment of *Salmonella* in pork products in Vietnam. The information generated in this study has been utilized in a risk assessment published recently [14], which showed that cross-contamination of *Salmonella* from raw to cooked pork at Vietnamese household was one of the most important factors influencing salmonellosis incidence.

## 2. Materials and Methods

### 2.1. Household Survey on Hygiene Management When Cooking Pork

In 2013, a total of 416 households in the Hung Yen and Nghe An provinces (208 in each province) were visited and interviews were conducted using a structured questionnaire on risk management practices when preparing pork. From these two provinces, three consumer areas representing rural, peri-urban and urban were selected for the study; from each study area, six towns and six rural communes were then included in the final research. Within the areas of study, households were randomly selected and one representative adult member (aged above 16) per household, usually the housewife, participated in the interview, as previously described by Nga et al. [25]. This interview included questions about diet, food access, food consumption, and food safety knowledge during handling practices under the pork safety project, PigRISK [26].

### 2.2. Preparation of Salmonella Culture

Previous studies have shown, *Salmonella* Typhimurium, *Salmonella* Derby and *Salmonella* London to be the most common serovars in pig carcasses and retailed pork in Vietnam [12,13,27]. These serovars of *S. enterica*, isolated from pig carcasses and retailed pork in a recent investigation by Dang-Xuan [27], were used to make an inoculation medium. First, each serovar was recovered and then cultured separately in a 150 mL-conical flask containing 50 mL Buffered Peptone Water (BPW) (Merck, Darmstadt, Germany) at 37 °C overnight (without agitation). The following day, *Salmonella* concentration was determined applying a plate count technique using Xylose Lysine Desoxycholate (XLD, Merck) agar for each cultured medium. Duplicate plates were made by spreading 0.1 mL of cultured BPW, diluted 10-fold with Maximum Recovery Diluent (MRD, Merck), on XLD plates and incubated at 37 °C for 20–24 h. After determining the *Salmonella* concentration, the culture was diluted to a concentration of 10^5^ CFU/mL. Following this 5 mL of medium containing each incubated serovar of *Salmonella* with 10^5^ CFU/mL were mixed, and 15 mL of the medium prepared. Then, MRD (90 mL) was added to 10 mL of the inoculated medium to achieve a concentration of 10^4^ CFU/mL medium (100 mL), which was then used to inoculate the pork.

### 2.3. Pork Preparation

Fresh cut pork was purchased immediately after splitting and deboning at a slaughterhouse in the early morning and prior to carcass transportation to the market. The sirloin and/or shoulders (containing both lean and fat areas) were selected. To minimize *Salmonella* contamination, sterile knives and gloves were used to cut the pork and remove the outer surface of the selected part without removing all subcutaneous and intermuscular fat. Twelve pork pieces (six sirloin and six shoulder pieces) weighing between 500 ± 40 g (approximately 14 × 6 × 5 cm ± 2 cm), from two different carcasses were cut and placed into individual sterile plastic bags and sealed. The pork specimens were kept cool and transported to the laboratory within three hours of collection to perform the experiments. At the laboratory, each pork piece was weighed and prepared for *Salmonella* inoculation.

### 2.4. Inoculation of Pork

Based on the weight of each pork piece, *Salmonella* culture (concentration of 10^4^ CFU/mL) was inoculated at a rate of 10 CFU/g of *S. enterica*. This concentration was based on a previous study measuring the *Salmonella* contamination range in marketed pork [27]. Approximately 500 ± 40 µL of the culture (1 µL for 1 g of the pork) was dispensed on the surface of the pork piece using a filter tip and pipette (Thermo Scientific, Madison, WI, USA). This covered the entire surface of the pork piece. The inoculated pork pieces were kept on a table at an ambient temperature (26–30 °C) for 30 min to allow cell attachment prior to starting the experiments as described by Ravishankar et al. [28].

### 2.5. Pork and Equipment Washing

Pork was washed twice in a basin with *Salmonella*-free water using bare hands. Washed pork then was placed on a cutting board and cut into 2–3 smaller pieces (approximately 150 g per piece, which is the size of 5 × 6 × 5cm ± 2cm). Washing of hands, knives and cutting boards was done separately using *Salmonella*-free water, dish-washing detergent and a dish cloth. Following washing, equipment was air dried for 75 min and then used in subsequent experimental steps. The boiled pork pieces were sliced into pieces of two to four millimeters thick with the length and width measuring were approximate two and five centimeters, respectively, as would be done when preparing pork for serving.

### 2.6. Sampling

Both hands (surface, palms, fingers, webbing), 25 cm^2^ of both sides of the knife and 25 cm^2^ of the cutting board surface were swabbed using sterile pre-moistened gauze. The surface samples from hands, knives, and cutting boards were collected immediately after washing raw pork twice and just before slicing cooked pork. Pork wash-water samples (approximately 30–40 mL per sample) were aseptically collected after twice washing the raw pork. Both raw and cooked pork samples were collected using sterile scalpels and forceps. Raw pork was sampled prior to pork being placed into a pot of boiling water. Cooked pork was sampled directly after boiling. The cooked pork slice was sampled immediately after slicing.

### 2.7. Design of Cross-Contamination Studies

**Figure 1:**
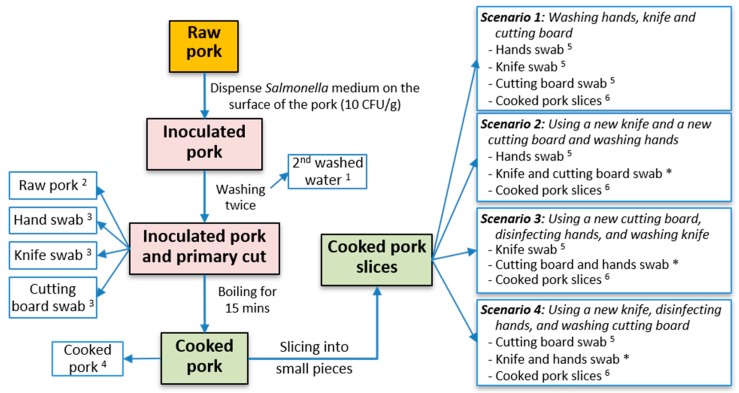
Steps and scenarios in experiment of *Salmonella enterica* cross-contamination. Timing associated with the steps: ^1^ after washing inoculated pork twice, ^2^ just before boiling after washing pork twice, ^3^ after primary cut of pork and washing once, ^4^ after boiling pork (wait until cool down) without any process, ^5^ just before slicing cooked pork, ^6^ after finishing slicing the cooked pork pieces, and * disinfection before slicing.

This study was designed to quantify the potential for transfer of *S. enterica* in home kitchens, from raw to cooked pork, via hands, knife (with a plastic handle and stainless-steel blade), and a wooden cutting board. The experimental design followed four main steps: (1) Raw pork was artificially inoculated with *S. enterica*; (2) The inoculated pork was then washed twice using *Salmonella*-free water (Lavie Ltd., Nestlé Water, DongNai, Vietnam); (3) It was then cut into smaller pieces and boiled in a pot with 2–2.5 L of water for 15 min; and (4) Following cooking, the pork was sliced. Four different preparation techniques (scenarios) were investigated based on cooking practice information obtained from the household survey (Figure 1). The *Salmonella* concentration in raw washed pork and occurrence of cross-contamination on hands, knives, and cutting boards was measured (Table 1). The contamination status and the level of *Salmonella* were also measured on the cooked pork. The experiment was carried out in triplicate in three groups, and repeated three times, equating to nine experimental trials for each scenario.

### 2.8. Cross-Contamination Scenarios

According to the household survey, all respondents typically washed pork, hands, knives and wooden cutting boards, but there were differences in whether separate knives and/or cutting boards were used for raw and cooked pork. Scenario 1, representing the most common practice reported in the household survey (see Section 3), examined the degree of cross-contamination when no separate knives and cutting boards were used. Scenarios 2–4 utilized different combinations of washing equipment and hands (Table 1). The details of scenario 1 are as follows: after the raw pork was washed and cut, the knife, cutting board and hands were washed in a basin with clean water at ambient temperature (26–30 °C) using dish-washing detergent (Sunlight, Unilever Co. Ltd., Ho Chi Minh, Vietnam) and a dish cloth (Suka, Luoisoi Co. Ltd., Ho Chi Minh, Vietnam) for approximately three minutes. All dish cloths used were autoclaved prior to the experiments. The washed knife and cutting board were then reused to slice the cooked pork by the same person. In scenario 2, after the washed raw pork was cut, hands were washed with clean water using dish-washing detergent and a dish cloth, and a new cutting board and new knife were used to slice cooked pork by the same person. In scenario 3, after washed raw pork was cut, the knife was washed in clean water using dish-washing detergent and a dish cloth, and hands were disinfected using both 70% ethanol (Con70, Tien Dung Co., Ltd., Ho Chi Minh, Vietnam) and instant hand sanitizer (Purell, Akron, OH, USA). Then a new cutting board and the washed knife were used to slice the cooked pork. In scenario 4, after the washed raw pork was cut, the cutting board was washed using clean water, dish detergent and a dish cloth, and hands were disinfected as described in scenario 3. Cooked pork was sliced on the washed cutting board using a new knife.

### 2.9. Microbiological Tests

*Salmonella* detection was carried out according to the ISO-6579: 2002 procedure [29]. In the pre-enrichment step, swabs or 10 mL of liquid samples were added up to 100 mL BPW for homogenization. Pork samples weighing 25 g were homogenized in 225 mL BPW. For *Salmonella* enumeration in pork samples, a 3 tube-Most Probable Number (MPN) method was used following ISO/TS-6579-2: 2012 [30]. In the pre-enrichment step of MPN, series of three tubes per dilution of 1–0.1–0.01 g and 10–1–0.1 g were prepared for the incubation of raw and cooked pork, respectively. Further steps of *Salmonella* detection and enumeration were previously described in Dang-Xuan [27].

### 2.10. Data Analysis and Modeling

All data were digitized in Excel 2010 (Microsoft, Redmond, WA, USA) spreadsheets. Descriptive statistics were performed using Chi-squared test and Fisher’s exact test to compare the proportions of samples contaminated with *Salmonella* using R version 3.3.2 (R Core team, Vienna, Austria, 2015).

To estimate the distributions of *Salmonella* concentration on pork slice in the scenarios, both non-parametric and parametric bootstrapping techniques were used. Bacterial concentration was measured as MPN/g (note that MPN/g and CFU/g hereafter is at the original bacterial count scale), and thus follows Log-Normal distribution with the mean MPN/g, and the standard deviation in log_10_ scale (*sd*_log10_) determined as shown in Equation (1) [31,32]:

(1) $$sd_{log10}=0.55\sqrt{\log_{10}\alpha }=0.55\sqrt{\log_{10}10}=0.55$$

where *α* is dilution ratio, ten. For the parametric bootstrapping in R, rlnorm(1, ln*μ*, *sd*_ln_) function was used to sample a single value from Log-Normal distribution, where ln*μ* is the natural logarithm of the MPN, and *sd*_ln_ is the standard deviation in the natural logarithm scale. *sd*_ln_ was calculated using natural logarithm (ln) as Equation (2):

(2) $$sd_{\ln}=\ln10^{sd_{\log10}}=\ln10^{0.55}=1.266422$$

In each scenario, a distribution of an MPN result was randomly selected at equal probability of selection among the MPN results of *Salmonella* positive samples for the type of the sample of interest. A value was randomly sampled from the distribution selected. For the mean MPN/g less than 0.03, a value was randomly selected from non-informative uniform distribution between natural logarithm of 0.01 MPN/g (−4.60517) and 0.03 MPN/g (−3.50656), and exponential of the value was calculated. This process was iterated 5000 times using a for-loop function written in R to obtain the integrated distribution for each scenario. The median, 2.5th and 97.5th values of the stored 5000 samples were obtained, and Log-Normal distribution was fit to the simulated sample data using a maximum likelihood method in fitdist() function in the fitdistrplus package [33] to obtain the mean and standard deviation. For the presentation of the distributions, kernel density was calculated in density() function using the simulated sample data and plotted using R.

The reduction rate in *Salmonella* CFU/g was modelled by dividing the value (CFU/g) sampled as above by 10 CFU/g, which gave the initial *Salmonella* concentration inoculated on the raw pork. The calculation of reduction rate was iterated 5000 times to determine distributions. The distribution of reduction rate was presented using a histogram.

As there were two MPN values which gave a result of 11 MPN/g in scenario 1 and 4, exceeding the inoculation level, above simulations on CFU/g and reduction rate distributions were performed without these MPN values and with these two values named worst case scenarios.

### 2.11. Ethical Statement

The experiments and *Salmonella* analysis were carried out at the Department of Veterinary Hygiene of National Institute of Veterinary Research (Hanoi, Vietnam). All volunteers gave informed consent for their participation in the study. Ethical approval of this study (No. 148/2012/YTCC-HD3) was obtained from the ethical committee of the Hanoi University of Public Health. This research was a part of the PigRISK project and the Taskforce for Food Safety Risk Assessment in Vietnam, funded by ACIAR [26].

## 3. Results

### 3.1. Household Survey

Most (87%) households reported that they washed their hands and equipment after handling raw pork with ambient temperature water, while the rest using hot water (Table 2). The most common practice in both provinces was to use the same knife and cutting board, washed in between, for both raw and cooked pork (71.4%, 297/416). Use of separate knives and cutting boards for raw and cooked pork was less common (16.1%, 67/416).

### 3.2. Effect of Washing Twice on the Prevalence and Salmonella Concentration in Raw Pork

**Figure 2:**
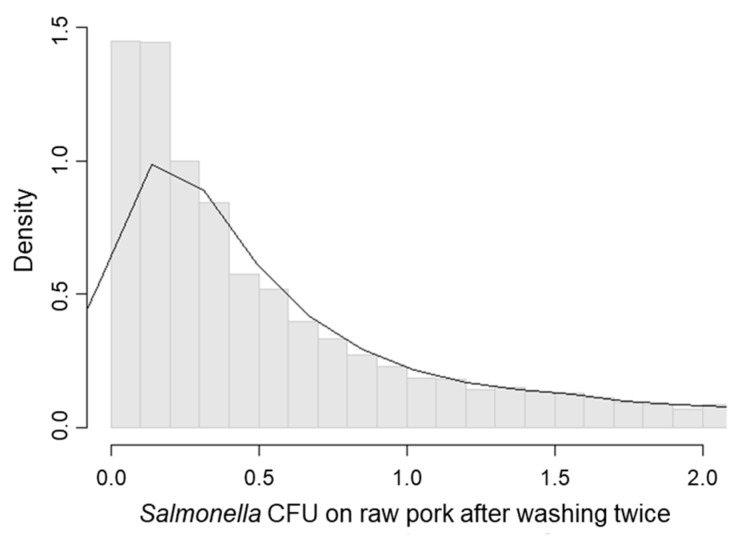
Distribution of *Salmonella* concentration (CFU/g) on raw pork after washing twice.

**Figure 3:**
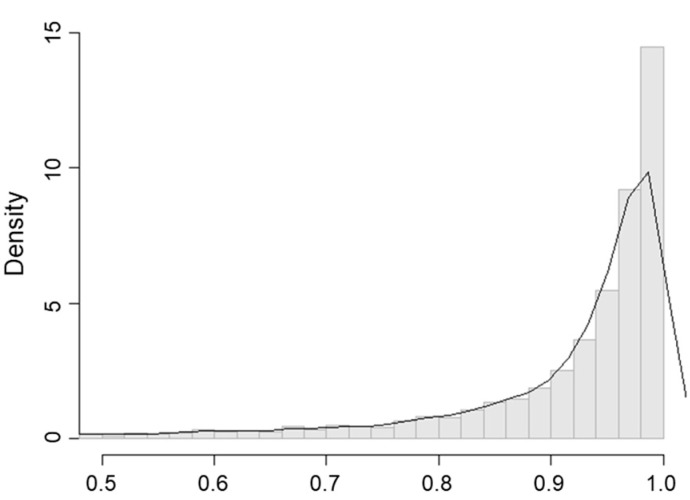
Reduction rate of *Salmonella* concentration in CFU/g after washing raw pork twice.

After twice washing raw pork, *Salmonella* was isolated from all nine samples with no reduction in prevalence observed. Table 3 shows the *Salmonella* concentrations of the raw pork samples. The simulated CFU/g of *Salmonella* in raw pork after washing twice was 1.56 (median 0.44; 95% CI: 0.03–10.14, Figure 2). Figure 3 shows the *Salmonella* concentration reduction rate measured on washed raw pork. The mean reduction rate of raw pork by washing twice in water was 84.4% (median 95.6%; 95% CI: −1.8–99.7%). These results suggested that washing raw pork can reduce bacteria levels but cannot eliminate *Salmonella* from the surface.

### 3.3. Cross-Contamination of Equipment and Hands with Salmonella from Raw Pork

Table 4 shows the proportions of sliced cooked pork, equipment, and hands for which *Salmonella* was transferred from raw to cooked pork (cross-contamination). Although raw pork was washed twice, cross-contamination with *Salmonella* from pork to hands, knives and cutting boards was common (78%, 78%, and 100%, respectively). Eight out of nine wash water samples were positive for *Salmonella*.

### 3.4. Re-Contamination of Cooked Pork Slices with Salmonella by Equipment and Hands

After cooking, *Salmonella* was not isolated from the nine pork samples. *Salmonella* was eliminated from pork by cooking, through re-contamination of boiled pork occurred in scenarios 1, 3, and 4 (Table 4). In scenario 2, new equipment (knife and cutting board) was used and re-contamination did not occur. The probability of re-contamination was highest in scenario 1, which did not involve use of separate equipment or disinfection of hands (7/9, 77.8%). There was no significant difference in the proportion of re-contamination between the scenarios re-using the cutting board after washing (scenarios 1 and 4, *p* = 1, Fisher’s exact test, Table 4). When the scenarios involving re-using the same cutting board were combined (1 and 4), the probability of re-contamination (72.2%) was higher than scenarios which used a new cutting board (scenarios 2 and 3) and where re-contamination was 11.1%. The difference between these proportions was found to be significant (*x*^2^ = 11.4, df = 1, *p* < 0.01).

**Figure 4:**
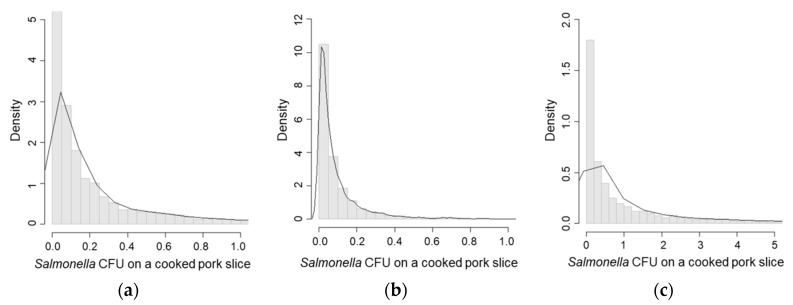
*Salmonella* CFU/g on a pork slice due to cross-contamination. (**a**) Scenario 1—Washing hands, the knife and the cutting board; (**b**) Scenario 3—Using a new cutting board, disinfecting hands, and washing the knife; (**c**) Scenario 4—Using a new knife, disinfecting hands, and washing the cutting board.

**Figure 5:**
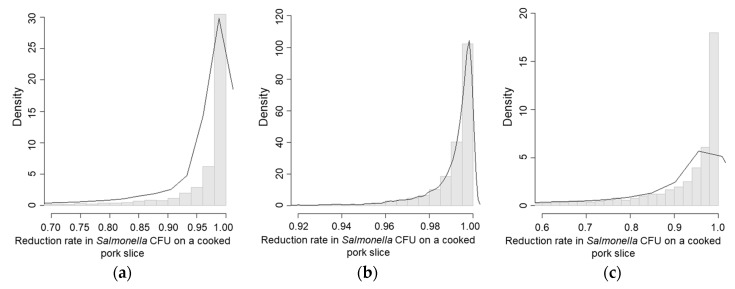
Reduction rates after *Salmonella* cross-contamination to cooked pork slice. (**a**) Scenario 1—Washing hands, the knife and the cutting board; (**b**) Scenario 3—Using a new cutting board, disinfecting hands, and washing the knife; (**c**) Scenario 4—Using a new knife, disinfecting hands, and washing the cutting board.

In scenario 4, (new knife, disinfected hands, and washed cutting board), the *Salmonella* concentration on cooked pork was the highest (mean CFU/g = 2.49, Table 3, Figure 4c) followed by the *Salmonella* concentration on cooked pork in scenarios 1 and 3 (Figure 4a,b). Scenario 4 also had the lowest reduction rate of *Salmonella* concentration on cooked pork (mean = 75.1%, Table 5, Figure 5c). Scenario 3, which represented the risk of re-contamination through re-use of a knife, showed low probability of re-contamination (mean = 22.2%, Table 4), and a higher reduction rate of *Salmonella* concentration (mean = 98.9%, Table 5, Figure 5b) compared with scenarios 1 and 4 (Figure 5a,c).

**Figure 6:**
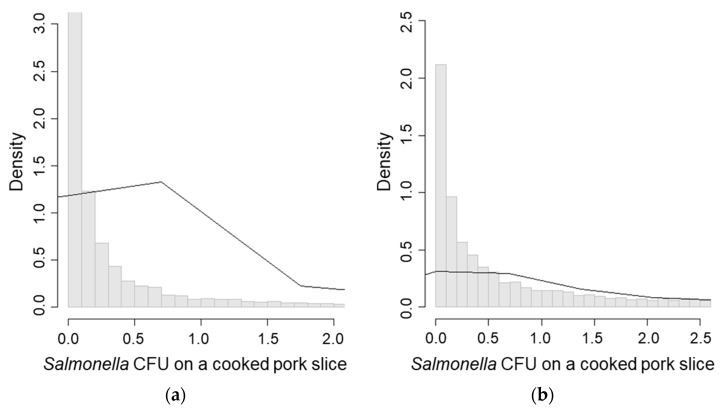
Worst case scenarios of *Salmonella* CFU/g on a pork slice due to cross-contamination. (**a**) Scenario 1—Washing hands, the knife and the cutting board; (**b**) Scenario 4—Using a new knife, disinfecting hands, and washing the cutting board. Worst case scenarios referred to the simulations on CFU/g and reduction rate distributions that were performed with two MPN values, which resulted as 11 MPN/g in scenario 1 and 4, exceeding the inoculation level.

In the two worst case scenarios (that were performed with two MPN values, which resulted as 11 MPN/g in scenario 1 and 4, exceeding the inoculation level), the mean *Salmonella* concentration re-contaminated on cooked pork in scenarios 1 (Figure 6a) and 4 (Figure 6b) were 4.21 CFU/g and 5.79 CFU/g (Table 3), respectively.

**Figure 7:**
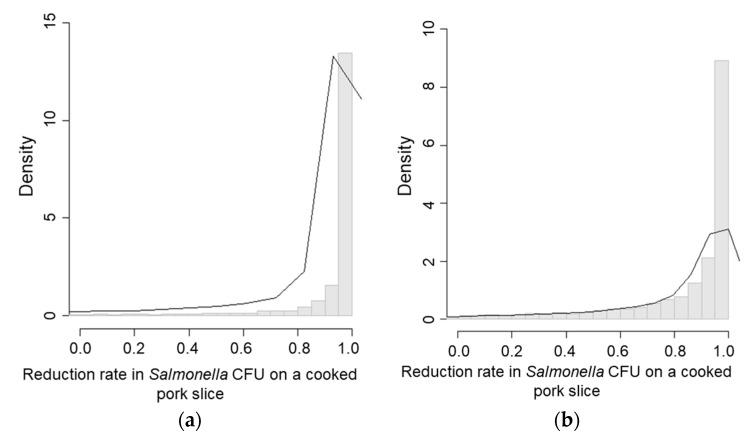
Reduction rate in CFU/g on a cooked pork slide in worst case scenarios. (**a**) Scenario 1—Washing hands, the knife and the cutting board; (**b**) Scenario 4—Using a new knife, disinfecting hands, and washing the cutting board. Worst case scenarios referred to the simulations on CFU/g and reduction rate distributions that were performed with two MPN values, which resulted as 11 MPN/g in scenario 1 and 4, exceeding the inoculation level.

The probability of cross-contamination in the worst case of scenarios 1 (Figure 7a) and 4 (Figure 7b) were almost 30% higher compared to their initial scenarios (Table 5). The probabilities of exceeding the initial CFU/g measurement in both scenarios 1 and 4 were 8.2% and 13.0%, respectively (Table 5).

## 4. Discussion

In this study, four different household food-handling behavior scenarios investigating *Salmonella* transmission were examined using cross-contamination experiments. The practices commonly used, supported by a field survey, were found to result in cross-contamination. In this experiment set, cross-contamination mainly occurred through use of same cutting board (scenarios 1 and 4). The practice of using the same utensil and/or cutting board to prepare both raw meats and other foods has been reported in the other countries: 25% to 83% of the respondents did this in the USA [21,34]. Such unsafe practices may cause cross-contamination during home food preparation.

The vast majority of salmonellosis cases have been linked to ingesting living *Salmonella* [35,36]. The results of this study suggest the significant contribution of cutting boards in the establishment of cross-contamination of *S. enterica*. The use of other utensils, knives and hands in home cooking processes are also known to play an important role in bacterial cross-contamination [37]. As such, washing of surfaces and equipment including cutting board and knives, and hands is reported to reduce bacterial contamination [38]. Use of detergent in washing kitchen equipment and hands also reduces transmission of diarrhea-causing pathogens [39] and is more effective than water alone [40]. However, the results of this study suggest that washing, even using dish detergent, has limited effect on elimination of *Salmonella* from the surfaces of kitchen equipment and hands (Scenario 1 and 4, 33.3–66.7%, Table 4). We used the same dish detergent for all washing steps, and other types of detergent such as hypochlorite [41] or organic acid [42,43] or hot water at 75 to 80 °C [43], as well as frequent and careful washing [41] may further reduce the chance of transmission of not only *Salmonella* but also other diarrhea-causing pathogens. Further, this study demonstrated that use of separate equipment between raw and cooked pork should also be encouraged.

The use of an autoclaved dishcloth for drying hands and utensils in this study means levels of cross-contamination was likely lower than real world enactment of these scenarios. Several studies have shown that kitchen dishcloths are often contaminated with bacteria and these would be an additional means of cross-contamination [44].

Remarkably, in the two worst case scenarios in this study, a higher *Salmonella* concentration than the initial inoculum was found after preparation, indicating microbial growth rather than reduction. An explanation for this may be that wooden cutting boards are known to absorb moisture which allows bacteria to adhere and multiply. Studies have shown that *Salmonella* can survive in deep cuts on wooden cutting boards [45,46] and that wood is one of the most difficult surfaces to disinfect [41]. Although washing cutting boards was a common practice in the studied areas, this experiment showed that bacteria can remain and be a source of cross-contamination. This finding has been reported in previous studies [47,48]. Therefore, future food safety intervention programs in Vietnam should focus on the risk of cross-contamination from cutting boards in home kitchens.

This study used a Bayesian approach, to present uncertainty and variability of *Salmonella* concentrations and reduction rates as probability distributions, using a limited number of samples. This information, together with the probability of cross-contamination in different hygiene procedures, is particularly useful in the exposure assessment step in risk assessment which usually lacks the data [20,49].

There are some limitations in this study. First, we took swabs from only 25 cm^2^ of each side of the knife and cooking board, which may underestimate cross-contamination. Second, we assumed no growth of *Salmonella* during the experiments. As this experiment included time to dry hands and equipment, which is not a common practice between people preparing food, the bacterial concentration presented in this study may be under-estimated. Third, the pork was not washed before it was inoculated with *Salmonella.* However, cut pork sampling at the slaughterhouse took place aseptically, so it is unlikely that significant *Salmonella* contamination would have occurred prior to inoculation and have affect the results. Forth, the sample size was relatively small and future study to increase the sample size would reduce the uncertainties in distributions. Fifth, the nature of the food, type of surfaces and level of moisture were reported as important factors influencing on microbial transfer rates [50,51]. Future studies may consider the conditions of time, temperature and surface type related to bacterial growth.

This study provided the first data on the possible occurrence and magnitude of cross-contamination which was used for quantitative risk assessment of *Salmonella* from household pork consumption in Vietnam [14]. The levels of cross-contamination in different scenarios will allow us to estimate potential risk-mitigating strategies. These findings may aid in promoting improvement in safer food handling practices in households, in addition to supporting risk communication and food safety education for consumers, and to minimize adverse health risk consequences [52,53]. The findings may counter the common misperception that if pork is cooked well before consumption it does not present a risk. The presence of *Salmonella* in ready-to-eat or cooked food due to cross-contamination has been reported in several studies [53,54] and the findings in our study can also be used for assessing the risks in these foods.

## 5. Conclusions

This study demonstrated that cross-contamination with *Salmonella* in household kitchens occurs when the same kitchen utensils (especially cutting boards) are used when preparing raw and cooked pork, even if they are washed between. This practice was common in households in Vietnam. On the contrary, no cross-contamination was observed when a different cutting board and knife was used for preparing raw and cooked pork, but this is rarely done in Vietnam. Radical changes in household cooking preparation may reduce the incidence of salmonellosis greatly in the country, and other parts of the world with similar settings. However, such changes may be difficult to promote, and risk reduction and adaptation of other options such as using difference types of cutting boards or different washing protocols should be examined.

## Figures and Tables

**Table 1:** Investigation of *Salmonella enterica* during cross-contamination experiments.

Sampling Points	Sample Type	Data
**Measuring remaining *Salmonella* levels after washing contaminated pork**		
Water used for washing	Water for washing recovered	Qualitative ^1^
Raw pork after being washed twice	Pork piece	MPN ^2^
**Testing whether cross-contamination with *Salmonella* occurs after cutting raw pork**		
Hands	Surface swab	Qualitative
Knife	Surface swab	Qualitative
Cutting board	Surface swab	Qualitative
**Ensuring *Salmonella* was inactivated**		
Cooked pork immediately after cooking	Pork piece	Qualitative
**Measuring the level of cross-contamination with *Salmonella* when handling the cooked pork in different scenarios**		
*Scenario 1: Washing hands, knife and cutting board*		
Hands before slicing	Surface swab	Qualitative
Knife before slicing	Surface swab	Qualitative
Cutting board before slicing	Surface swab	Qualitative
Pork after slicing	Pork slice	MPN
*Scenario 2: Using a new knife and a new cutting board and washing hands*		
Hands before slicing	Surface swab	Qualitative
Pork after slicing	Pork slice	MPN
*Scenario 3: Using a new cutting board, disinfecting hands, and washing knife*		
Knife before slicing	Surface swab	Qualitative
Pork after slicing	Pork slice	MPN
*Scenario 4: Using a new knife, disinfecting hands, and washing cutting board*		
Cutting board before slicing	Surface swab	Qualitative
Pork after slicing	Pork slice	MPN

^1^ Presence or absence of *Salmonella* in 25 g pork tested; ^2^ Quantifying the number of *Salmonella* using 3-tube Most Probable Number (MPN) estimation method.

**Table 2:** Use of separate equipment between raw and cooked pork, and water temperature for washing hands and equipment after handling raw pork in cooking pork slice in households in Hung Yen and Nghe An provinces.

Handling Practices at Households	Hung Yen (n = 208, Frequency, %)	Households in Nghe An (n = 208, Frequency, %)	Overall (n = 416, Frequency, %)
Use of separate knife and cutting board between raw and cooked pork			
Separate knives and separate cutting boards were not used	141 (67.8)	156 (75.0)	297 (71.4)
Separate knives and separate cutting boards were used	36 (17.3)	31 (14.9)	67 (16.1)
Separate cutting boards were used, but separate knives were not used	18 (8.6)	18 (8.6)	36 (8.7)
Separate knives were used, but separate cutting boards were not used	11 (5.3)	2 (1.0)	13 (3.1)
Answer not provided	2 (1.0)	1 (0.5)	3 (0.7)
Water temperature for washing hands, knife, cutting board after handling raw pork			
Ambient temperature water with dishwashing detergent	191 (91.8)	169 (81.2)	360 (86.5)
Hot water (40–60 °C) with dishwashing detergent	15 (7.2)	39 (18.8)	54 (13.0)
Answer not provided	2 (1.0)	0 (0.0)	2 (0.5)

**Table 3:** Frequencies of MPN/g results categorized in MPN ranges and integrated *Salmonella* concentration distributions as mean, median, and confidence interval of CFU/g for washed raw pork and cooked pork slice in different cooking scenarios.

Scenario	Number of Samples in *Salmonella* MPN/g ^1^ Ranges				Mean CFU/g ^2^ (median)	95% CI
	<0.03	0.03–0.30	0.31–3.0	>3.0		
Raw pork after washing twice	0	1	8	0	1.56 (0.44)	0.03–10.14
Cooked pork slice						
Scenario 1	1	4	1	0	0.71 (0.12)	0.00–5.96
Scenario 1 ^3^	1	4	1	1	4.21 (0.16)	0.00–40.20
Scenario 3	1	1	0	0	0.12 (0.05)	0.00–0.67
Scenario 4	0	3	2	0	2.49 (0.44)	0.01–17.78
Scenario 4 ^3^	0	3	2	1	5.79 (0.71)	0.01–47.06

^1^ Most Probable Number, ^2^ Colony Forming Unit, ^3^ Scenarios referred to the simulations on CFU/g and reduction rate distributions that were performed with two MPN values, which resulted as 11 MPN/g in scenario 1 and 4, exceeding the inoculation level. Scenario 1—Washing hands, the knife and the cutting board, Scenario 3—Using a new cutting board, disinfecting hands, and washing the knife, Scenario 4—Using a new knife, disinfecting hands, and washing the cutting board.

**Table 4:** Proportions of cooked pork slice, hands, equipment and wash water on which cross-contamination with *Salmonella* occurred during the experiments.

Type of Sample	Samples Cross-Contaminated (n = 9)	Proportion Contaminated (%)	95% CI
*After raw pork handling*			
Hands	7	77.8	40.2–96.1
Knife	7	77.8	40.2–96.1
Cutting board	9	100	62.9–100
Wash water	8	88.9	50.7–99.4
*Scenario 1 ^1^*			
Cooked pork slice	7	77.8	40.2–96.1
Hands	3	33.3	9.0–69.1
Knife	4	44.4	15.3–77.3
Cutting board	5	55.6	22.7–84.7
*Scenario 2*			
Cooked pork slice	0	0.0	0.0–37.1
Hands	3	33.3	9.0–69.1
*Scenario 3*			
Cooked pork slice	2	22.2	3.9–59.8
Knife	0	0.0	0.0–37.1
*Scenario 4*			
Cooked pork slice	6	66.7	30.9–90.9
Cutting board	6	66.7	30.9–90.9

^1^ Scenario 1—Washing hands, the knife and the cutting board, Scenario 3—Using a new cutting board, disinfecting hands, and washing the knife, Scenario 4—Using a new knife, disinfecting hands, and washing the cutting board.

**Table 5:** Reduction rate of *Salmonella* concentration.

Scenario	Mean Reduction Rate (%)	Median (%)	Lower Limit (%)	Upper Limit (%)	Exceeded Initial CFU/g (%) ^1^
Scenario 1	92.7	98.8	44.9	99.9	1.0
Scenario 1 ^2^	57.9	98.4	−308.1	99.9	8.2
Scenario 3	98.9	99.5	93.8	99.9	0
Scenario 4	75.1	95.6	−78.0	99.9	5.2
Scenario 4 ^2^	42.1	92.9	−372.6	99.9	13.0

^1^ Colony Forming Unit, ^2^ Scenarios referred to the simulations on CFU/g and reduction rate distributions that were performed with two MPN values, which resulted as 11 MPN/g in scenario 1 and 4, exceeding the inoculation level. Scenario 1—Washing hands, the knife and the cutting board, Scenario 3—Using a new cutting board, disinfecting hands, and washing the knife, Scenario 4—Using a new knife, disinfecting hands, and washing the cutting board.
